# 2531. Pharmacokinetic-Pharmacodynamic (PK-PD) Target Attainment Analyses to Support Ceftobiprole Dosing Regimens for Patients with *Staphylococcus aureus* Bacteremia (SAB)

**DOI:** 10.1093/ofid/ofad500.2149

**Published:** 2023-11-27

**Authors:** Sujata M Bhavnani, Jeffrey P Hammel, Anthony J Rinaldo, Jennifer Smart, Karine Litherland, Leonard R Duncan, Mark E Jones, Marc Engelhardt, Paul G Ambrose, Christopher M Rubino

**Affiliations:** Institute for Clinical Pharmacodynamics, Schenectady, NY; Institute for Clinical Pharmacodynamics, Schenectady, NY; Institute for Clinical Pharmacodynamics, Schenectady, NY; Basilea Pharmaceutica International Ltd, Allschwil, Basel-Landschaft, Switzerland; Basilea Pharmaceutica International Ltd, Allschwil, Basel-Landschaft, Switzerland; JMI Laboratories, North Liberty, Iowa; Basilea Pharmaceutica International Ltd., Allschwil, Switzerland, Allschwil, Basel-Landschaft, Switzerland; Basilea Pharmaceutica International Ltd., Allschwil, Switzerland, Allschwil, Basel-Landschaft, Switzerland; Institute for Clinical Pharmacodynamics, Schenectady, NY; Institute for Clinical Pharmacodynamics, Schenectady, NY

## Abstract

**Background:**

Ceftobiprole medocaril is an intravenously (IV) administered cephalosporin that is rapidly converted to the active moiety ceftobiprole *in vivo*. Ceftobiprole medocaril is currently being developed for the treatment of patients with SAB, including infective endocarditis. PK-PD target attainment analyses were performed in order to provide support for ceftobiprole dosing regimens for the treatment of patients with SAB.

**Methods:**

Using a previously developed population PK model for ceftobiprole, %T > MIC targets for efficacy, and *in vitro* surveillance data for *S. aureus*, percent probabilities of PK-PD target attainment were evaluated for ceftobiprole dosing regimens among simulated patients with SAB. Simulated patients by creatinine clearance group (CLcr) and resembling the clinical trial population of the ERADICATE Phase 3 study [IDWeek 2022, LB202] were generated using replication of these clinical trial data. Dosing regimens by CLcr group that matched drug exposures to ceftobiprole 500 mg IV every 6 hours on Days 1 to 8 and every 8 hours on Day 9 and onwards among simulated patients with normal renal function were identified and then assessed among simulated patients. Randomly assigned ceftobiprole free-drug plasma %T > MIC targets associated with net bacterial stasis and a 1-log_10_ CFU reduction from baseline for *S. aureus* based on data from a neutropenic murine-thigh infection model were assessed.

**Results:**

Selected ceftobiprole dosing regimens for patients with SAB by CLcr group are shown in **Table 1**. **Table 2** shows the percent probabilities of PK-PD target attainment by MIC value and overall on Days 1 and 10. The highest MIC values at which percent probabilities of PK-PD target attainment were ≥ 90% based on free-drug plasma %T > MIC targets associated with net bacterial stasis and a 1-log_10_ CFU reduction from baseline for *S. aureus* were ≥ 4 µg/mL (i.e., the MIC value inhibiting 100% all *S. aureus* isolates, including MRSA) among simulated patient groups. Overall percent probabilities of PK-PD target attainment were > 99.9%.

**Figure:**
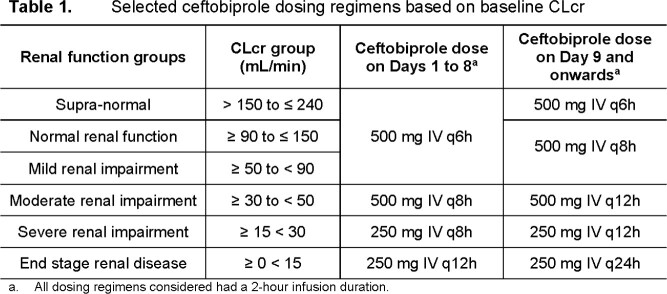

**Figure:**
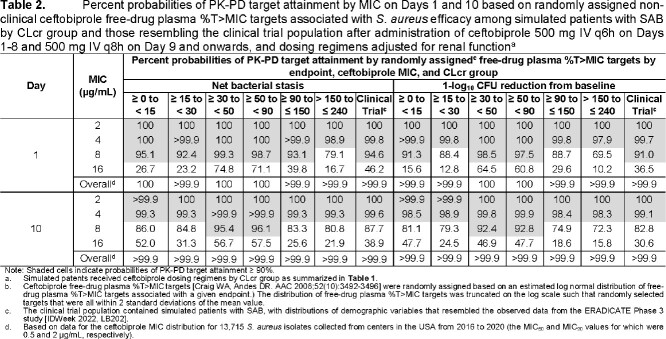

**Conclusion:**

Results of PK-PD target attainment analyses provided support for ceftobiprole dosing regimens for patients with SAB with 500 mg IV every 6 hours on Days 1 to 8 and every 8 hours from Day 9. Dosing regimens were adjusted for renal function.

**Disclosures:**

**Sujata M. Bhavnani, PharmD; MS; FIDSA**, Adagio Therapeutics, Inc.: Grant/Research Support|Albany Medical Center: Grant/Research Support|Amplyx Pharmaceuticals, Inc.: Grant/Research Support|AN2 Therapeutics: Grant/Research Support|Antabio SAS: Grant/Research Support|Arcutis Biotherapeutics, Inc.: Grant/Research Support|B. Braun Medical Inc.: Grant/Research Support|Basilea Pharmaceutica: Grant/Research Support|BioFire Diagnostics LLC: Grant/Research Support|Boston Pharmaceuticals: Grant/Research Support|Cidara Therapeutics Inc.: Grant/Research Support|Cipla USA: Grant/Research Support|Crestone Inc.: Grant/Research Support|CXC: Grant/Research Support|Debiopharm International SA: Grant/Research Support|Entasis Therapeutics: Grant/Research Support|Genentech: Grant/Research Support|GlaxoSmithKline: Grant/Research Support|Hoffmann-La Roche: Grant/Research Support|ICPD: Ownership Interest|Inotrem: Grant/Research Support|Insmed Inc.: Grant/Research Support|Iterum Therapeutics Limited: Grant/Research Support|Kaizen Bioscience, Co.: Grant/Research Support|KBP Biosciences USA: Grant/Research Support|Matinas Biopharma: Grant/Research Support|Meiji Seika Pharma Co., Ltd.: Grant/Research Support|Melinta Therapeutics: Grant/Research Support|Menarini Ricerche S.p.A.: Grant/Research Support|Mutabilis: Grant/Research Support|Nabriva Therapeutics AG: Grant/Research Support|Paratek Pharmaceuticals, Inc.: Grant/Research Support|Qpex Biopharma: Grant/Research Support|Sfunga Therapeutics: Grant/Research Support|Spero Therapeutics: Grant/Research Support|Suzhou Sinovent Pharmaceuticals Co.: Grant/Research Support|Theravance: Grant/Research Support|tranScrip Partners: Grant/Research Support|University of Wisconsin: Grant/Research Support|Utility Therapeutics: Grant/Research Support|ValanBio Therapeutics Inc.: Grant/Research Support|VenatoRx: Grant/Research Support **Jeffrey P. Hammel, MS**, Adagio Therapeutics, Inc.: Grant/Research Support|Albany Medical Center: Grant/Research Support|Amplyx Pharmaceuticals, Inc.: Grant/Research Support|AN2 Therapeutics: Grant/Research Support|Antabio SAS: Grant/Research Support|Arcutis Biotherapeutics, Inc.: Grant/Research Support|B. Braun Medical Inc.: Grant/Research Support|Basilea Pharmaceutica: Grant/Research Support|BioFire Diagnostics LLC: Grant/Research Support|Boston Pharmaceuticals: Grant/Research Support|Cidara Therapeutics Inc.: Grant/Research Support|Cipla USA: Grant/Research Support|Crestone Inc.: Grant/Research Support|CXC: Grant/Research Support|Debiopharm International SA: Grant/Research Support|Entasis Therapeutics: Grant/Research Support|Genentech: Grant/Research Support|GlaxoSmithKline: Grant/Research Support|Hoffmann-La Roche: Grant/Research Support|ICPD: Employee|Inotrem: Grant/Research Support|Insmed Inc.: Grant/Research Support|Iterum Therapeutics Limited: Grant/Research Support|Kaizen Bioscience, Co.: Grant/Research Support|KBP Biosciences USA: Grant/Research Support|Matinas Biopharma: Grant/Research Support|Meiji Seika Pharma Co., Ltd.: Grant/Research Support|Melinta Therapeutics: Grant/Research Support|Menarini Ricerche S.p.A.: Grant/Research Support|Mutabilis: Grant/Research Support|Nabriva Therapeutics AG: Grant/Research Support|Paratek Pharmaceuticals, Inc.: Grant/Research Support|Qpex Biopharma: Grant/Research Support|Sfunga Therapeutics: Grant/Research Support|Spero Therapeutics: Grant/Research Support|Suzhou Sinovent Pharmaceuticals Co.: Grant/Research Support|Theravance: Grant/Research Support|tranScrip Partners: Grant/Research Support|University of Wisconsin: Grant/Research Support|Utility Therapeutics: Grant/Research Support|ValanBio Therapeutics Inc.: Grant/Research Support|VenatoRx: Grant/Research Support **Anthony J. Rinaldo, B.S.**, Adagio Therapeutics, Inc.: Grant/Research Support|Albany Medical Center: Grant/Research Support|Amplyx Pharmaceuticals, Inc.: Grant/Research Support|AN2 Therapeutics: Grant/Research Support|Antabio SAS: Grant/Research Support|Arcutis Biotherapeutics, Inc.: Grant/Research Support|B. Braun Medical Inc.: Grant/Research Support|Basilea Pharmaceutica: Grant/Research Support|BioFire Diagnostics LLC: Grant/Research Support|Boston Pharmaceuticals: Grant/Research Support|Cidara Therapeutics Inc.: Grant/Research Support|Cipla USA: Grant/Research Support|Crestone Inc.: Grant/Research Support|CXC: Grant/Research Support|Debiopharm International SA: Grant/Research Support|Entasis Therapeutics: Grant/Research Support|Genentech: Grant/Research Support|GlaxoSmithKline: Grant/Research Support|Hoffmann-La Roche: Grant/Research Support|ICPD: Employee|Inotrem: Grant/Research Support|Insmed Inc.: Grant/Research Support|Iterum Therapeutics Limited: Grant/Research Support|Kaizen Bioscience, Co.: Grant/Research Support|KBP Biosciences USA: Grant/Research Support|Matinas Biopharma: Grant/Research Support|Meiji Seika Pharma Co., Ltd.: Grant/Research Support|Melinta Therapeutics: Grant/Research Support|Menarini Ricerche S.p.A.: Grant/Research Support|Mutabilis: Grant/Research Support|Nabriva Therapeutics AG: Grant/Research Support|Paratek Pharmaceuticals, Inc.: Grant/Research Support|Qpex Biopharma: Grant/Research Support|Sfunga Therapeutics: Grant/Research Support|Spero Therapeutics: Grant/Research Support|Suzhou Sinovent Pharmaceuticals Co.: Grant/Research Support|Theravance: Grant/Research Support|tranScrip Partners: Grant/Research Support|University of Wisconsin: Grant/Research Support|Utility Therapeutics: Grant/Research Support|ValanBio Therapeutics Inc.: Grant/Research Support|VenatoRx: Grant/Research Support **Jennifer Smart, PhD**, Basilea Pharmaceutica International Ltd, Allschwil, Switzerland: Stocks/Bonds **Karine Litherland, Ph.D.**, Basilea Pharmaceutica International Ltd, Allschwil, Switzerland: Full time employee|Basilea Pharmaceutica International Ltd, Allschwil, Switzerland: Stocks/Bonds **Leonard R. Duncan, PhD**, AbbVie: Grant/Research Support|Basilea: Grant/Research Support|CorMedix: Grant/Research Support|Melinta: Grant/Research Support|Pfizer: Grant/Research Support **Mark E. Jones, PhD**, Astellas Pharma Global Development, Inc: Support for the present publication|Basilea Pharmaceutica International Ltd: Employee of Basilea Pharmaceutica International Ltd|Basilea Pharmaceutica International Ltd: Stocks/Bonds **Marc Engelhardt, MD**, Astellas Pharma Global Development, Inc.: Support for the present publication|Basilea Pharmaceutica International Ltd: Employee of Basilea Pharmaceutica International Ltd|Basilea Pharmaceutica International Ltd: Stocks/Bonds **Paul G. Ambrose, PharmD; MS; FIDSA**, Adagio Therapeutics, Inc.: Grant/Research Support|Albany Medical Center: Grant/Research Support|Amplyx Pharmaceuticals, Inc.: Grant/Research Support|AN2 Therapeutics: Grant/Research Support|Antabio SAS: Grant/Research Support|Arcutis Biotherapeutics, Inc.: Grant/Research Support|B. Braun Medical Inc.: Grant/Research Support|Basilea Pharmaceutica: Grant/Research Support|BioFire Diagnostics LLC: Grant/Research Support|Boston Pharmaceuticals: Grant/Research Support|Cidara Therapeutics Inc.: Grant/Research Support|Cipla USA: Grant/Research Support|Crestone Inc.: Grant/Research Support|CXC: Grant/Research Support|Debiopharm International SA: Grant/Research Support|Entasis Therapeutics: Grant/Research Support|Genentech: Grant/Research Support|GlaxoSmithKline: Grant/Research Support|Hoffmann-La Roche: Grant/Research Support|ICPD: Ownership Interest|Inotrem: Grant/Research Support|Insmed Inc.: Grant/Research Support|Iterum Therapeutics Limited: Grant/Research Support|Kaizen Bioscience, Co.: Grant/Research Support|KBP Biosciences USA: Grant/Research Support|Matinas Biopharma: Grant/Research Support|Meiji Seika Pharma Co., Ltd.: Grant/Research Support|Melinta Therapeutics: Grant/Research Support|Menarini Ricerche S.p.A.: Grant/Research Support|Mutabilis: Grant/Research Support|Nabriva Therapeutics AG: Grant/Research Support|Paratek Pharmaceuticals, Inc.: Grant/Research Support|Qpex Biopharma: Grant/Research Support|Sfunga Therapeutics: Grant/Research Support|Spero Therapeutics: Grant/Research Support|Suzhou Sinovent Pharmaceuticals Co.: Grant/Research Support|Theravance: Grant/Research Support|tranScrip Partners: Grant/Research Support|University of Wisconsin: Grant/Research Support|Utility Therapeutics: Grant/Research Support|ValanBio Therapeutics Inc.: Grant/Research Support|VenatoRx: Grant/Research Support **Christopher M. Rubino, PharmD**, Adagio Therapeutics, Inc.: Grant/Research Support|Albany Medical Center: Grant/Research Support|Amplyx Pharmaceuticals, Inc.: Grant/Research Support|AN2 Therapeutics: Grant/Research Support|Antabio SAS: Grant/Research Support|Arcutis Biotherapeutics, Inc.: Grant/Research Support|B. Braun Medical Inc.: Grant/Research Support|Basilea Pharmaceutica: Grant/Research Support|BioFire Diagnostics LLC: Grant/Research Support|Boston Pharmaceuticals: Grant/Research Support|Cidara Therapeutics Inc.: Grant/Research Support|Cipla USA: Grant/Research Support|Crestone Inc.: Grant/Research Support|CXC: Grant/Research Support|Debiopharm International SA: Grant/Research Support|Entasis Therapeutics: Grant/Research Support|Genentech: Grant/Research Support|GlaxoSmithKline: Grant/Research Support|Hoffmann-La Roche: Grant/Research Support|ICPD: Ownership Interest|Inotrem: Grant/Research Support|Insmed Inc.: Grant/Research Support|Iterum Therapeutics Limited: Grant/Research Support|Kaizen Bioscience, Co.: Grant/Research Support|KBP Biosciences USA: Grant/Research Support|Matinas Biopharma: Grant/Research Support|Meiji Seika Pharma Co., Ltd.: Grant/Research Support|Melinta Therapeutics: Grant/Research Support|Menarini Ricerche S.p.A.: Grant/Research Support|Mutabilis: Grant/Research Support|Nabriva Therapeutics AG: Grant/Research Support|Paratek Pharmaceuticals, Inc.: Grant/Research Support|Qpex Biopharma: Grant/Research Support|Sfunga Therapeutics: Grant/Research Support|Spero Therapeutics: Grant/Research Support|Suzhou Sinovent Pharmaceuticals Co.: Grant/Research Support|Theravance: Grant/Research Support|tranScrip Partners: Grant/Research Support|University of Wisconsin: Grant/Research Support|Utility Therapeutics: Grant/Research Support|ValanBio Therapeutics Inc.: Grant/Research Support|VenatoRx: Grant/Research Support

